# Physical activity correlates in young women with depressive symptoms: a qualitative study

**DOI:** 10.1186/1479-5868-7-3

**Published:** 2010-01-18

**Authors:** Denise Azar, Kylie Ball, Jo Salmon, Verity J Cleland

**Affiliations:** 1 Centre for Physical Activity and Nutrition Research, School of Exercise and Nutrition Sciences, Deakin University, 221 Burwood Hwy, Burwood, VIC, 3125, Australia

## Abstract

**Background:**

Young women are at high risk for developing depression and participation in physical activity may prevent or treat the disorder. However, the influences on physical activity behaviors of young women with depression are not well understood. The aim of this study was to gather in-depth information about the correlates of physical activity among young women with and without depressive symptoms.

**Methods:**

A sample of 40 young women (aged 18-30 years), 20 with depressive symptoms (assessed using the CES-D 10) and 20 without depressive symptoms participated in one-on-one semi-structured interviews. A social-ecological framework was used, focusing on the individual, social and physical environmental influences on physical activity. Thematic analyses were performed on transcribed interview data.

**Results:**

The results indicated several key themes that were unique to women with depressive symptoms. These women more often described negative physical activity experiences during their youth, more barriers to physical activity, participating in more spontaneous than planned activity, lower self-efficacy for physical activity and being influenced by their friends' and family's inactivity.

**Conclusions:**

Interventions designed to promote physical activity in this important target group should consider strategies to reduce/overcome early life negative experiences, engage support from family and friends and plan for activity in advance.

## Background

Globally, depression is the fourth leading cause of disease burden [1]. In 2003, depression and anxiety disorders were ranked the second leading cause of burden of disease and injury in Australia [2]. Young adults are a population group at high risk of experiencing depression [3,4] and are also a group at increased risk of declining physical activity levels [5-7]. Although young adults are generally more active than mid-aged and older-adults, evidence suggests that the steepest decline in physical activity occurs during late adolescence and young adulthood [8], particularly amongst women. Furthermore, women in young adulthood participate in lower levels of physical activity than men [9,10]. In addition to its benefits to physical health, many studies indicate that participating in physical activity can also have positive effects on psychological health, such as reducing risk or symptoms of depression [11,12]. A recent review [13] found that physical activity reduces depressive symptoms and even low levels of activity can prevent symptoms among young women. While, we have a clearer understanding on the association between physical activity and depressive symptoms among young women, the underlying factors that influence physical activity behavior in these women compared to women without symptoms are unknown. To increase physical activity participation among young women with depressive symptoms, it is important to identify modifiable influences on these behaviors.

Social-ecological models suggest that an individual's physical activity is influenced by a diverse range of individual, social and physical environmental factors [14]. One study examined psychosocial correlates of physical activity in women (aged 18-65 years) with depressive symptoms [15]. This study found that low self-efficacy and social support for activity and barriers such as fatigue and a lack of time were evident. To date, there has been no research examining the correlates of physical activity in young women with depressive symptoms. Research with young women within the general population has identified early life experiences of physical activity [16], individual barriers [17,18] such as lack of time and fatigue, enjoyment [19,20], motivation [21] and self-efficacy [22,23] as being associated with physical activity participation. Social correlates of physical activity amongst young women include social support from family and friends [20,24] and physical environmental correlates include having greater access to sporting facilities in the local neighborhood and sporting equipment in the home [25,26].

Although research in the general population suggests that physical activity is inversely associated with depression [11], the majority of studies have been cross-sectional and several reviews have concluded that more evidence is needed to determine temporal effects/causal nature of this well-established association. Depression might be associated with physical activity if for instance a depressed mood state leads to a lack of interest in motivation for physical activity. Alternatively, physical activity participation might influence risk of depression by impacting on either physiological or psychological characteristics associated with mood state.

This study presents the findings of a series of in-depth one-on-one interviews conducted to explore perceptions of physical activity and its determinants in young women with and without depressive symptoms. Based on the Social Ecological Model [14], this qualitative study aims to gather in-depth information to provide insights into the individual, social and physical environmental correlates of physical activity among a sample of young women with and without depressive symptoms. This study also aimed to explore the possible direction of the relationship between physical activity and mood/depression among these women.

## Methods

This study involved in-depth interviews with 40 women aged 18-30 years. A qualitative research design has the advantage of providing detailed contextual insights into the influences on physical activity and the relationship between depression and physical activity from the perspectives of young women that might not otherwise emerge from a quantitative method [27]. Qualitative research allows more detailed explanations of temporal/causal directions of influence. Data were collected between April and May 2008. The study was approved by the Deakin University Human Research Ethics Committee, and participants provided informed written consent to take part.

### Recruitment

Recruitment strategies included promotion of the study on the website research noticeboard for "beyondblue", an Australian national not-for-profit mental health initiative. Advertising material was also posted around the Burwood campus of Deakin University (Melbourne, Australia), and on the university website at the Burwood campus and two campuses in Geelong (Australia). Deakin University is a public university and the Burwood campus is attended by more than 16,000 students, and the two Geelong campuses by approximately 4,500 students. Snowball sampling was also employed. This is a technique whereby existing participants are asked if any acquaintances may also be interested in participating in the study and information is forwarded to potential participants through current participants [28]. After interviews had been conducted with 20 women with depressive symptoms and 20 without symptoms, it was evident that little new information was forthcoming, therefore it was considered that data saturation was reached and recruitment of participants ceased. To be eligible for inclusion in this study, participants had to be female, aged between 18 and 30 years, and not currently pregnant. Five women did not meet the criteria with one being pregnant and four not in the age range of interest.

### Materials

The Centre for Epidemiologic Studies Depression Scale (CES-D) is a 20-item self-report scale used to identify the persistence and severity of depressive symptoms among the general population [29]. The 10-item CES-D 10 [30] is comparable to a clinical diagnosis in depressed patients and is as reliable as the original CES-D instrument [31]. Participants were asked to indicate how often in the past week they experienced 10 feelings (e.g. "I felt that everything I did was an effort" and "I felt lonely"). Responses were summed across the 10 items to provide a total score ranging from zero to 30. Based on the CES-D 10 protocol/recommendations [30], a score of 10 or more indicated the participant had depressive symptoms and participants were classified as such. Participants who scored below 10 points were classified as not having depressive symptoms.

A semi-structured interview schedule was developed based on the Social Ecological Model [14] of the individual, social and physical environmental influences on physical activity (Table 1). The interview schedule included broad topic areas derived from previous research [13,17,19-22,25]. The interview schedule was piloted and refined with the first five participants with only minor changes made for clarity. Physical activity was defined for participants at the beginning of the interview and questions relating to the four domains of physical activity (i.e. leisure-time, transport-related, household, work-related) were discussed, with particular focus on leisure-time activity. At the commencement of the interview a brief self-report questionnaire was administered that included variables such as age, height, weight, country of birth, marital status, highest educational qualification and employment status. This information was used for descriptive purposes only.

**Table 1:** Interview schedule of questions

Topic area	Questions
Individual influences	• Can you describe your physical activity as a child and adolescent?
	• How much physical activity do you currently do?
	• What types of physical activities do you do for leisure/work/household/transport?
	• How do these physical activities make you feel?
	• Do you want to do more physical activity?
	• What are the main factors that may prevent you from being as active as you'd like?
	• If you're feeling tired/stressed/unwell, does this impact on your physical activity in your leisure-time?
	• How confident are you that you can overcome these feelings at the time and still be active in your leisure-time?
	• Does your mood predict how active you are?
	• What helps/motivates you to be active?
	• Did you became less active because of your mental health or did you develop depression because you were inactive?
	• Who are you active with in your leisure-time?
	• Is it a different experience for you when you are active with someone rather than alone?
Social influences	• How active are the people around you?
	• Do they encourage you to be active?
	• Does anyone try to stop you from being active?
	• Does their physical activity behaviours influence yours?
	• How do you perceive the sporting and recreational facilities in your neighbourhood?
Physical environmental influences	• Where are you active in your leisure-time?
	• Thinking about these places, what things are important to you being physically active there?
	• What things do you think would help you to be more physically active around your neighbourhood?

### Data collection

Potential participants contacted a trained female researcher by email or telephone, where a full explanation of the study was provided. Those who met the inclusion criteria then completed the CES-D 10 via telephone and were classified as 'with depressive symptoms' or 'without depressive symptoms'. Participants were informed of their depressive symptom status prior to the interview. Those classified as having depressive symptoms were assured that this only meant they were at risk of depression, it was not a clinical diagnosis, and referral/treatment suggestions were provided where necessary. A convenient and private time and location was arranged for the interview, for example, the participants' university library, which was conducted by the same researcher.

This same researcher individually interviewed the participants and the interviews lasted between 20 and 45 minutes. All interviews were audio-taped with the participants' permission. At the end of the interview, participants were asked if there was anything else they would like to discuss. Those who had symptoms of depression were offered referral advice/information should they wish to seek assistance or support. Participants were provided with a cinema voucher as compensation for their time.

### Data management and analysis

Each interview was transcribed verbatim and the transcribing checked by the first author. Using raw transcripts, a manual qualitative data analysis method outlined by Green et al. [32] was employed. This involved four key steps: immersion in the data, coding, creating categories, and the identification of themes. Immersion in the data was achieved by reading and re-reading of the interview transcripts and listening to the interview recordings. Coding of the transcripts was conducted by creating and assigning descriptive labels to segments of the transcript. Data were entered into NVivo [33] to facilitate coding and for the creation of categories and sub-categories and extraction of quotes. Creating categories involved grouping codes that shared similar content. The identification of themes was the final step of data analysis and involved giving interpretation to the data and referring to theoretical concepts relevant to the topic, based on the constructs of the Social Ecological Model. Quotes were selected to demonstrate responses which were common or which represented a concise summary of a topic.

## Results

Twenty-one women were recruited from university and 19 were recruited from the general population. More women without depressive symptoms worked full-time and had a tertiary education compared to women with symptoms (Table 2). The majority of women with symptoms of depression were university students. Two broad categories of themes were investigated when analysing the data: themes that were based on the aims of this study, termed 'expectant themes'; and themes which were not intentionally sought and became apparent during the interview process, termed 'emergent themes' [34]. Ten broad themes that were described differently across the two groups were identified. The expectant themes included early life physical activity experiences; the relationship between physical activity and depression; motivations for physical activity; barriers to physical activity; encouragement/support for physical activity; social norms for physical activity; the social context of physical activity and the perception of recreational facilities in the physical environment. The emergent themes that were identified included planning for physical activity and stress-relief. These themes are described below.

**Table 2:** Socio-demographic characteristics of participants

	With depressive symptoms n = 20	Without depressive symptoms n = 20
	**M**	**M**
**Age**	23.4	25.4
**Body mass index**	23.8	22.5
**Education**	**N**	**N**
Some school/completed school	9	4
Tertiary (TAFE, diploma, university, post-graduate)	11	16
**Employment**		
Working full-time	5	14
Studying full-time	12	6
Studying part-time	3	0
**Marital status**		
Single	14	12
De facto/living together	4	3
Married	2	4
Separated/widowed/divorced	0	1

### Early life physical activity experiences

A number of women with depressive symptoms mentioned that while growing up, their family or parents were not active in their leisure time and that these experiences had influenced their current physical activity behaviors. There were also suggestions from women with depressive symptoms that they felt resentful of the fact that physical activity in the family environment was not encouraged while growing up. In contrast, women without depressive symptoms more often mentioned that their family was active during their youth and that one or both parents participated in sporting groups or clubs. This is illustrated in the following quote:

*I grew up in a really active household. My mum and dad were very active. My mum went to aerobics almost every day and I remember going there as a small child *(Woman without depressive symptoms, 26 years).

Women with depressive symptoms also more frequently described having negative physical activity experiences as a child or adolescent. These women tended to avoid situations that involved physical activity or sports while growing up, although they commonly pointed out that if one particular sport that they liked was on offer, they would participate (see quote below). The main reasons for avoiding physical activity were a lack of enjoyment of school sports, self-consciousness in a classroom setting, perceived lack of confidence to perform physical activities or lack of encouragement to participate. More women with depressive symptoms than women without mentioned that they were not active as a child and/or adolescent. Conversely, almost all of the women without symptoms stated that they were highly active as a child and moderately to highly active as adolescents, and they mostly discussed positive early life physical activity experiences. The following quotes demonstrate the contrast in the two groups' perceived early life physical activity experiences.

*I wasn't really a big fan of sport at primary school. Sort of tried to avoid it when I could. We had a person come and talk to us once from the local squash courts and gave us a bit of a demonstration. I enjoyed that. And I joined up to the squash club near my home and I sort of went along...But I wasn't in that for too long...maybe a couple of months *(Woman with depressive symptoms, 22 years).

*As a child, I loved playing all sports. We did PE [physical education] at school which was probably my favorite time of the week, I played basketball and netball a few times a week *(Woman without depressive symptoms, 24 years).

### Relationship between physical activity and depression/mood

Participants were asked whether they believed there was a relationship between their mood and physical activity. More specifically, participants were asked to explain whether they felt that their mood influenced their physical activity behavior or vice versa. Women with depressive symptoms generally responded that their mood influenced their physical activity behavior.

*If I'm a bit more upbeat I'm more likely to go to the gym, whereas if I'm feeling down, tired or stressed...I'm less likely to do exercise. If I get up and I've got a bit of energy, I think 'I'll get myself to the gym today' *(Woman with depressive symptoms, 18 years).

When women with depressive symptoms discussed times in their life when they experienced severe depression, they reported that their physical activity decreased. Several women with depressive symptoms described how their depression affected all aspects of their life and that undertaking day-to-day activities was a struggle, resulting in physical activity not being a priority.

... *I had been quite unwell for a long time and so...the main issue was to sort out the depression. Because it was even a struggle to go to work and stuff at that point *(Woman with depressive symptoms, 29 years).

Women without depressive symptoms tended to believe that their physical activity behavior predicted their mood, more than the reverse.

*I think my mood's better when I do [physical activity]. I do find it relaxing. It kind of clears my thoughts if I go out for a walk or for a run *(Woman without depressive symptoms, 23 years).

### Motivations for physical activity

Among women with depressive symptoms, weight control and experiencing health benefits were most often mentioned as motivators. Weight control was a motivation for activity, either to assist with losing weight or maintaining weight, while a small number of women also mentioned that receiving compliments on their figure when they lost weight was a motivating factor. Other benefits from physical activity were commonly mentioned as motivators, including being fit, feeling good about oneself and seeing results. The following is a quote from a woman with depressive symptoms regarding her motivations for physical activity:

*Being complimented. So if someone says 'wow you're looking healthy!' and also just feeling a lot better. Like I got up the stairs today and wasn't killing myself. Definitely just seeing the results of doing activity and getting some external kind of reinforcement as well *(Woman with depressive symptoms, 29 years).

Women without depressive symptoms also mentioned having company and experiencing benefits as motivators for physical activity. Weight control was not as commonly reported as a motivator by women without symptoms, however, enjoyment of physical activity was more often reported among these women. This quote represents a typical response from women without depressive symptoms:

*I guess I like to stay fit...I also just enjoy physical activity. I do actually like playing in a team, that's good *(Woman without depressive symptoms, 23 years).

Among both groups of women, lack of motivation was commonly cited as a reason that stopped them from being as active as they would like to be. A few women also alluded to or admitted that they were lazy when it comes to being active.

*Stopping me [from being active]? I'm just lazy I think. I'm not motivated. I'll get really motivated every now and then, that's when I go for a run, but then other days I'll just be like 'I'll do it tomorrow' *(Woman with depressive symptoms, 21 years).

### Barriers to physical activity

#### - Lack of time

Lack of time was a commonly mentioned barrier to being active noted by both groups of women. The reasons causing lack of time mainly focused around study or work commitments. Among women with depressive symptoms, family/household responsibilities and distance to university/work were also cited as a cause for lack of time for physical activity participation.

#### - Low priority for physical activity

For several of the women with depressive symptoms who spoke of lack of time as a barrier to physical activity, it appeared that low priority for physical activity was an important underlying factor. Other commitments, such as university work, held higher priority for their time than physical activity. This was not a barrier expressed by women without depressive symptoms.

*Pretty much the study's the priority because one, it's costing me and two, it's what I'm passionate about...so I want to get that done and then physical activity will come next *(Woman with depressive symptoms, 26 years).

Other women with depressive symptoms expressed that they felt guilty engaging in physical activity because they perceived other commitments to be equally, if not more important.

*I think, oh what should I do this afternoon? Should I go for a walk or should I do this reading that I need to have done by tomorrow? It's like, well, I should do the work *(Women with depressive symptoms, 22 years).

#### - Making excuses/procrastination

Among women with depressive symptoms, it emerged that procrastination and making excuses were reasons for not engaging in physical activity. This was not an evident barrier among women without depressive symptoms. Some women with depressive symptoms had formed a habit of attending to a number of other things instead of participating in physical activity, while others described making excuses as to why they could not include physical activity in their day-to-day activities.

*I'll procrastinate on the internet for a half a day thinking to myself I've got to do the dishes. And then just before I have to go somewhere else I'll do the dishes so I don't actually have time to do exercise in between. So it's just kind of making excuses and not actually making the time to do it *(Woman with depressive symptoms, 26 years).

#### - Low self-efficacy

Low self-efficacy for physical activity was more commonly described by women with depressive symptoms. The majority of these women mentioned that they were not very confident in engaging in physical activity in difficult situations including when feeling tired, stressed and unwell.

*If I'm tired I can usually say to myself, oh well, don't go for a walk today; yeah it's pretty easy to forgo the walk or the yoga/Pilates class *(Woman with depressive symptoms, 25 years).

In contrast, the majority of women without depressive symptoms discussed being quite confident in their ability to be active under difficult circumstances. However, some of these women did mention that feeling tired or unwell may cause them to reduce the time or intensity of their physical activity.

### Planning for physical activity

Physical activity often seemed to be more spontaneous and less planned for women with depressive symptoms than for those without symptoms. Women without depressive symptoms expressed that if they planned to engage in physical activity, they would most likely follow through on their plan. However, for women with symptoms their physical activity behavior depended on their state of mind at the time.

*Yeah sometimes it's hard to just step out and go 'well this is what you're going to do...' It really depends on the moment *(Woman with depressive symptoms, 26 years).

*More often than not, if I'm going to go for a run or a swim, I plan it the day before say get my stuff ready and so for me the planning most often means that I'll go *(Woman without depressive symptoms, 23 years).

### Stress-relief

Women from both groups, but particularly those without depressive symptoms, believed that they would be more likely to be active when they were stressed. They commonly believed that when they felt stressed, physical activity produced stress-relief benefits and a clear mind.

*If I'm feeling stressed I'll actually push myself to go to the gym more because as people say you have to exercise to get the stress out and release your happy hormones *(Woman with depressive symptoms, 21 years).

*If I'm feeling stressed it would [be] more likely that I will go and do it, because I will always know that afterwards I will feel better *(Woman without depressive symptoms, 29 years).

However, some women with depressive symptoms expressed choosing not to exercise despite knowing the benefits of being active when stressed.

*Yeah, that [stress] would affect me too, so I'm likely to binge eat or just sit at home rather than actually going out to do something. Even though the exercise would be a stress relief *(Woman with depressive symptoms, 26 years).

### Social correlates

#### Social support for physical activity

##### Encouragement/support for physical activity

Most women from both groups mentioned that they receive encouragement or support for physical activity from members of their family, friends and partners, but mainly family, which usually came from their mothers.

*Well she'll [mum] say 'let's play tennis, let's go out, go running'. She's been on to me to do sports *(Woman with depressive symptoms, 19 years).

##### Social norms for physical activity

Women in both groups described being influenced by significant others' physical activity. However, women without depressive symptoms more often reported being positively influenced by others being active. They also more frequently mentioned that they received motivation from people in their lives who were inactive.

*I think when I'm around people who aren't active, it kind of motivates me to be more active. Because I just can't stand laziness and people not exercising. So, it just motivates me to do the right thing for my body. When I'm around people who are active, that motivates me even more to keep active *(Woman without depressive symptoms, 28 years).

Women with depressive symptoms commonly described being positively influenced by others' being active, but, in contrast to women without depressive symptoms, if they knew inactive people, they were negatively influenced by their inactivity.

*People who are active I guess make me feel bad when I'm not being active, because I feel I should be doing that. People who are non-active, probably have the same effect. If they're not active I think, 'oh they're not doing it', so I can have a rest too or something *(Woman with depressive symptoms, 21 years).

##### Social context for physical activity

Women with depressive symptoms more frequently described being active alone than with others. They also commonly mentioned that being active with others could be both a positive and negative experience. These women gave a number of reasons for preferring to be active alone, including others not being able to keep up with their pace, preferring walking on their own to think things through, and disliking the pressure to perform at other people's level. In contrast, women without depressive symptoms mentioned only positive experiences in being active with others. Namely, physical activity with others was described as more enjoyable, more motivating and made them push themselves to exercise a bit harder or longer.

### Physical environmental correlates

#### The perception of recreational facilities in the physical environment

When asked what facilities and supports around the neighborhood would help them to be more active, several women with depressive symptoms expressed that there was nothing in their physical environment that could help.

*Because I'm not a gym goer and I don't play sport usually, my main physical activity has been walking and so that very much depends just on my frame of mind more than any environment stuff *(Woman with depressive symptoms, 29 years).

Other women with depressive symptoms mentioned that having more facilities such as tennis courts, walking tracks and a dog park may make them more active. Affordability of facilities, neighborhood safety features such as lighting and footpaths were also mentioned by these women. Women without depressive symptoms most commonly reported wanting more facilities for physical activity in their neighborhood such as tennis courts and walking tracks, community initiatives such as free gym and boot camp trials, advertising about available facilities in the local neighborhood and the need for greater affordability of facilities, particularly at local gyms.

## Discussion

The results indicate that women with depressive symptoms more often described negative physical activity experiences during their youth, perceived physical activity as a lower priority, more often mentioned low self-confidence in being active, described slightly different motivators to be active, were less likely to describe planned (as opposed to spontaneous) physical activity, and that those close to them who were inactive influenced them to be inactive too, compared to women without symptoms of depression.

One of this study's aims was to explore the perceived direction of the relationship between physical activity and depressive symptoms. Women without depressive symptoms expressed that physical activity influenced their mood more than the reverse. On the other hand, women with depressive symptoms more often reported that their mood influenced their physical activity behavior. It is not surprising that women without depressive symptoms talked less often about their mood influencing their physical activity because they are less likely to have experienced depressed mood states that may impact on their decisions to be active. The findings suggest that women with depressive symptoms may be less active because their feelings of depression strongly influence whether or not they participate in physical activity. This is consistent with previous research which showed that mood disturbance is negatively associated with physical activity among adults, in that people who experience a mood disorder, such as depression, are consequently less likely to be active [35]. It may be important that strategies for promoting physical activity amongst those at risk of depression consider the additional challenge of mood state.

The importance of early life physical activity experiences was a key theme that emerged from the interviews. Women with depressive symptoms more frequently described being less active as a child and adolescent than women without symptoms. Whilst it is not clear whether women in the current study have experienced depressive symptoms since childhood, past research has shown that less physically active children and adolescents are more likely to experience depressive symptoms [36,37]. In this study, those with depressive symptoms more commonly mentioned growing up in an inactive household and having negative experiences of physical activity as a child and adolescent. Being exposed to few positive physical activity opportunities in their youth may have lead to the development of mental health problems such as depression. It should be acknowledged that the women's retrospective recall of negative early experiences of physical activity may be adversely influenced by the women's current low mood state and/or current low physical activity levels, in that they may want to attribute their low activity levels to negative early life experiences. Nevertheless, since physical activity during young adulthood may predict later physical activity participation among women [38,39], it is important that any negative perceptions of physical activity be addressed early to prevent these perceptions from contributing to lower participation and potential risk of depression later in life.

Lack of time and lack of motivation were commonly reported as barriers to physical activity irrespective of depressive status, consistent with past research in women [40]. Perceived self-efficacy for physical activity was described differently by women with and without depressive symptoms. Women with depressive symptoms described having low confidence in their ability to be active in adverse situations, while women without symptoms tended to express high confidence. Bandura [41] argued that lack of self-efficacy for physical activity was associated with greater symptoms of depression. When people engage in physical activity and perceive improvements (e.g. fitness gains, bodily changes), self-efficacy may improve [41]. As a result, the person feels better about themselves and hence depressive symptoms may reduce. Therefore, exploring strategies that focus on increasing self-efficacy for physical activity among young women with depressive symptoms is important.

The role of planning for physical activity was an emergent theme arising from the interviews. Women without depressive symptoms more frequently expressed that their physical activity behavior was planned in advance, whereas the physical activity behavior of women with depressive symptoms often appeared to be more spontaneous in nature. A construct of the Theory of Planned Behavior suggests the effort people devote towards planning physical activity may directly predict participation [42]. A recent study found that perceived effort which included planning and similar constructs of willpower, energy, trying and discipline, was the most important factor in determining physical activity participation among young adults [43]. In that study, perceived effort predicted physical activity behavior more so than intentions, attitudes, subjective norms, perceptions of control and past behavior. Given that effort and planning are important for physical activity participation and that it is more likely to occur if it is planned, women with depressive symptoms may increase their activity if they actively plan for physical activity in their day-to-day activities; for example, by preparing their exercise clothes or equipment the night before.

Stress-relief from physical activity was also a theme that emerged from the interviews. Women without depressive symptoms expressed that physical activity was a behavior they engaged in when they felt stressed as a means to reduce their stress levels, which is consistent with research suggesting physical activity is effective in reducing stress levels among adults [44]. However, several women with depressive symptoms mentioned that they did not engage in physical activity when they felt stressed, which may subsequently increase their depressive symptoms given the close relationship between stress and depression [45]. Depressive symptoms can also lead to feelings of listlessness and lethargy [46], which in combination with feelings of stress may result in an even lower desire to participate in physical activity. These women may choose to try undesirable means in order to reduce stress, such as smoking and alcohol, which are observed more frequently among individuals who experience depression than in the general population [47,48]. Alternatively, physical activity programs may reduce stress levels through incorporation of adaptive coping strategies such as counseling.

Women with and without symptoms of depression perceived high levels of support and encouragement for physical activity from family and friends in this study. These findings contradict studies that have shown women with depressive symptoms to have lower social support in general [49] and for physical activity [15] than women without symptoms of depression. Studies have shown that social support for physical activity by family and friends is a strong predictor of being active amongst young women [20,24]. Furthermore, a study of young adults found high social support was associated with being physically active irrespective of participants' depression status [50]. While both groups of women were positively influenced by significant others' activity, women with symptoms of depression also expressed being negatively influenced by others' inactivity. These findings highlight the role of positive social networks and could reflect how individuals with depression are often strongly influenced by those in their lives, especially when the influence provides the option of avoiding tasks perceived to be less important or difficult, such as physical activity [51].

In the present study, the majority of women with depressive symptoms reported that no amount of facilities or supports in the physical environment would make them more active. They acknowledged that their local neighbourhood had good facilities, but that they lacked the motivation or interest to access them. This appears to contradict past research that suggests good facilities are associated with being active [25]. The findings suggest that the physical environment may not be as important in influencing physical activity among young women with depressive symptoms. Previous research [52,53] examining the influences on the individual, social and physical environments on physical activity found that a combination of all levels of influence contribute to explaining physical activity behavior, although the participants in these studies did not have depressive symptoms.

There are limitations of this study that should be considered when interpreting the findings. Selection bias may be a limiting factor because participants may have had a keen interest in the areas of physical activity and mental health, and therefore may be more aware of the health benefits or facilities in their area. Using a university as one of the recruitment sites has potential implications, given that university students have a higher level of education than people in the general population, and they are likely to have other unique characteristics. Further research in other population groups such as people of low socio-economic position including low- or middle-income countries may produce different findings. A possible limitation of this study was that the interviewer administered the CES-D 10 prior to the interview and therefore was aware of the participants' depression status. This knowledge may have influenced the direction of questioning; however, the same interviewer conducted all interviews and the same questions were asked of all participants. Also, participants' awareness of their depression status may have resulted in socially desirable responses; though, all participants were forthcoming in reporting positive and negative behaviors irrespective of depression status, so this seems unlikely. Additionally, the information provided by participants in the interviews are perceived accounts rather than objective reports, and social desirability may have resulted in participants providing more positive responses regarding their physical activity behaviors than was actually the case. However, there seemed to be variation in responses with many women reporting that they were 'lazy' and not doing sufficient physical activity for their health.

Despite these limitations, the in-depth interview methodology is a valuable technique for gaining information that may not be obtained through quantitative methods, such as rich contextual data on beliefs, values and experiences. A strength of this study was the investigation of a range of individual, social and physical environmental influences under the guidance of a Social Ecological Model. Furthermore, no previous study has specifically explored these issues among young women with depressive symptoms. Whilst the present findings suggest common elements, there are also some key differences in the potential influences amongst women with and without depressive symptoms. Consequently, "blanket" approaches for physical activity promotion among young women are not likely to be effective; rather, tailored strategies for young women with depressive symptoms are needed. These may include engaging social support from family and friends in a community walking group program, and overcoming early life physical activity negative experiences by increasing competence in sports and activities through skill building. Strategies to increase self-efficacy may include daily monitoring of physical activity and reward systems, and planning for physical activity through encouragement of visual and physical reminders may be helpful to perform more activity. Further research is required to obtain a better understanding of the relationship between physical activity and depression among young women. Particularly, greater knowledge about the temporal effects of this relationship is important to identify the appropriate time of intervention; therefore qualitative longitudinal research may be a novel approach to address gaps in knowledge in this target group.

## Abbreviations

CES-D 10: Center for Epidemiological Studies Depression Scale 10-item

## Competing interests

The authors declare that they have no competing interests.

## Authors' contributions

DA carried out data collection, conducted data analyses and led the writing of the manuscript. KB, JS and VJC participated in the design of the study and helped to draft the manuscript. All the authors read and approved the final manuscript.
